# Orthopaedic Surgical Demand Index: A Measure of Need in the United States

**DOI:** 10.5435/JAAOSGlobal-D-22-00131

**Published:** 2022-11-15

**Authors:** Maxwell Davison-Kerwood, Sam Jiang, Mark Gonzalez1

**Affiliations:** From the Department of Orthopedic Surgery (Davison-Kerwood), University of Illinois, and the University of Illinois College of Medicine (Jiang), Chicago, IL.

## Abstract

Before being evaluated by a physician, more than 50% of patients will search their symptoms on the Google search engine. In fact, Google was the engine used for nearly 90% of all online searches between 2016 and 2018. These search data are stored by Google and can be investigated through google extended trends for health (GETH). The goal of this research was to use GETH to correlate Google search probabilities for elective orthopaedic procedures with the orthopaedic surgeon density in each US state to create a surgical demand index (SDI) that could be then compared between states. In addition, this study aims to assess the effects of annual income, percent minority population, and unemployment rate on that SDI.

**Methods:**

Google search probabilities were collected using the Google Trends Extraction Tool. Search probabilities were collected in each state for composite search terms. Data were collected in monthly intervals between 2016 and 2018 and averaged. The states were grouped into geographic regions. One-way analysis of variance and pairwise Mann-Whitney *U* tests were done between these regions. Linear regression analysis was conducted to assess the effect of median annual statewide income, percent minority population, and unemployment rate with SDI.

**Results:**

The analysis of variance and Mann-Whitney *U* tests demonstrated a difference between regions. Linear regression analysis revealed a notable effect of median income on SDI, but no effect of percent minority population or unemployment rate.

**Conclusions:**

The Midwest and South had higher regional demand than the Northeast and West, with West Virginia being the most in need and the District of Columbia being the least in need. Annual median income had a notable negative effect on SDI, whereas percent minority population and unemployment rate had no effect. This study highlights the inequality that exists in the southern and midwestern United States and identifies one potential predictive factor of this unequal SDI.

Before being evaluated by a physician, more than 50% of patients will search their medical symptoms on the Google search engine.^1^ In fact, between 2016 and 2018, nearly 90% of online searches in the United States (US) were done using Google, the world's largest search engine.^2^ The relative search volume for specific search terms and Google-defined topics can be accessed by an open-access platform provided by Google called Google Trends, an application programming interface (API). A closed-access version of the platform exists for healthcare researchers called Google Extended Trends API for Health (GETH), which provides search probabilities—a more statistically accurate and malleable version of the data—rather than relative search volumes. Google Trends and GETH data research have become widely used in a field of research termed “infodemiology,” which uses Big Data to investigate public health and public policy.^3^ Highlighting the rapid growth of infodemiology, research using Google Trends and GETH has risen 20-fold from 2009 to 2018.^3^ Within health care, these tools have been used to detect influenza hotspots faster than the Center for Disease Control, record increased depression-related searches during specific hours of the day, discover trends in public interest for specific surgical procedures, and much more.^4-6^

Google search data for surgical terms, i.e., “total knee arthroplasty,” have been positively correlated with the actual number of subsequent procedures performed.^6^ This provides support for the basis of this study, which sets out to use GETH data to correlate Google search probabilities for elective orthopaedic procedures with the orthopaedic surgeon density in each US state to create a surgical demand index (SDI) that can be compared between states. This novel SDI will enable researchers and clinicians to identify which states are hypothetically underserved using a more nuanced metric than just surgeon density by taking into consideration the component of public interest. This concept was investigated for the field of plastic surgery, but no such literature exists for orthopaedics.^7,8^ In addition, this study aims to assess the effects of annual income, percent minority population, and unemployment rate on SDI. Should a relationship exist between these metrics, it could point to states with greater demand for outpatient orthopaedic care and help uncover the reasons driving disparities and identify inequalities in health care across the United States. Although many other metrics could be investigated, these were decided to be the focus of this study.

## Methods

Google search probabilities were collected using the Google Trends Extraction Tool, a novel user interface for querying the GETH API.^9^ This tool increases data accuracy by compiling multiple samples taken from the Google database into a single, more accurate data point. GETH reports the search probability of each search term within the indicated time and geographical area. Search probabilities were collected in each state for the composite search terms “knee surgery + knee replacement + knee arthroplasty,” “hip surgery + hip replacement + hip arthroplasty,” “shoulder surgery + shoulder replacement + shoulder arthroplasty + rotator cuff repair + rotator cuff surgery,” and “back surgery + spine surgery + spinal surgery.” The Google Trends Extraction Tool failed to retrieve values for Maryland and Montana, so the GETH Python API was used to retrieve data for those two states using identical query terms and search intervals.^10^ 30 samples were taken and averaged to improve the accuracy for each data point in the Google Trends Extraction Tool, while a single sample was taken for each data point in the GETH Python API due to inherent limitations of the API.

Search probabilities were collected in monthly intervals between 2016 and 2018 (n = 36) and averaged to create a mean search probability value for each state. Three years were chosen to reduce the effect of random variation from year to year without capturing the effects of long-term change in interest. Data from 2019 and beyond were excluded to avoid any confounding volatility of public interest due to the COVID-19 pandemic, which began internationally toward the end of 2019.^11,12^

The means of each composite query were summed to create a cumulative “interest score” in each state (Equation 1). The interest score was then divided by the number of orthopaedic surgeons per 100,000 population, obtained from the 2018 American Academy of Orthopaedic Surgeons (AAOS) census, to generate a SDI for each state,^13^

(1) $$I_{Google}=\overset{N}{\underset{n=1}{\sum }}{\left(T_{1}+T_{2}+\ldots +T_{i}\right)}_{n}.$$

Surgical Interest Score (*I*_*Google*_) for a given state. *N* is the number of composite queries, and *T*_i_ is the mean search probability for each search term across the study period (*i.e.* the interest for the composite “knee surgery” search terms during all months of the study period was summed),

(2) $$D_{Ortho}=\frac{I_{Google}\times N_{Population}}{100000\times N_{Ortho}}.$$

Orthopaedic SDI (*D*_*Ortho*_) for a given state. *I*_*Google*_ is the surgical interest score, *N*_*Population*_ is the state population, and *N*_*Ortho*_ is the number of orthopaedic surgeons in the state.

The results were grouped into four geographic regions: West, Midwest, South, and Northeast.^14^ One-way analysis of variance and pairwise Mann-Whitney *U* tests were done to compare mean SDI between each region. Ordinary linear regression analysis was conducted to assess the effect of median annual statewide income, percent minority population, and unemployment rate with SDI.^14^ Statistical significance was set to 0.05 for all analyses. Data analysis was done using Python version 3.8.8.

## Results

**Figure 1:**
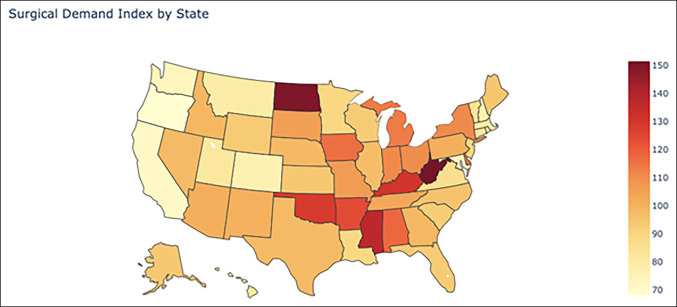
Map showing the orthopaedic surgeon density in the United States, defined as the number of orthopaedic surgeons per 100,000 population.

**Figure 2:**
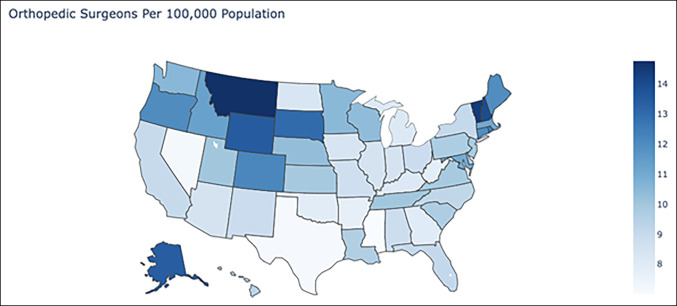
Map showing the geographical representation of the Surgical Demand Index (SDI) in the United States, with higher intensity indicating greater surgical demand. SDI is defined as the Google search interest score per orthopaedic surgeon per 100,000 population.

The mean and median SDI across the United States were 98.13 and 95.33, respectively, with a standard deviation of 19.41. West Virginia had the highest SDI of 151.44, and the District of Columbia had the lowest SDI of 60.26. Mississippi, North Dakota, and West Virginia were greater than 2 standard deviations above the mean, while Alabama, Arkansas, Kentucky, and Oklahoma were greater than 1 standard deviation above the mean. California, Colorado, District of Columbia, Hawaii, Maryland, Montana, New Hampshire, Oregon, Rhode Island, and Washington were greater than 1 standard deviation below the mean, and no states were greater than 2 standard deviations below the mean. The number of surgeons per 100,000 people is shown in Figure 1, and the heatmap of the SDI is shown in Figure 2. The average SDIs of the region are given in Table 1, and of each state and the District of Columbia in supplemental table 1, http://links.lww.com/JG9/A245.

**Table 1:** Mean SDIs for Each US Geographical Region

Region	Mean (SD)	Minimum (State)	Maximum (State)
Northeast	89.47 (10.51)	78.01 (New Hampshire)	111.29 (New York)
Midwest	106.27 (15.18)	89.47 (Minnesota)	150.04 (North Dakota)
South	106.3 (23.68)	60.26 (District of Columbia)	151.44 (West Virginia)
West	85.91 (11.47)	68.25 (Oregon)	101.02 (Arizona)

SDI = surgical demand index

The states in each region were assigned according to the US Census Bureau Statistical Groupings of States and Counties.

**Figure 3:**
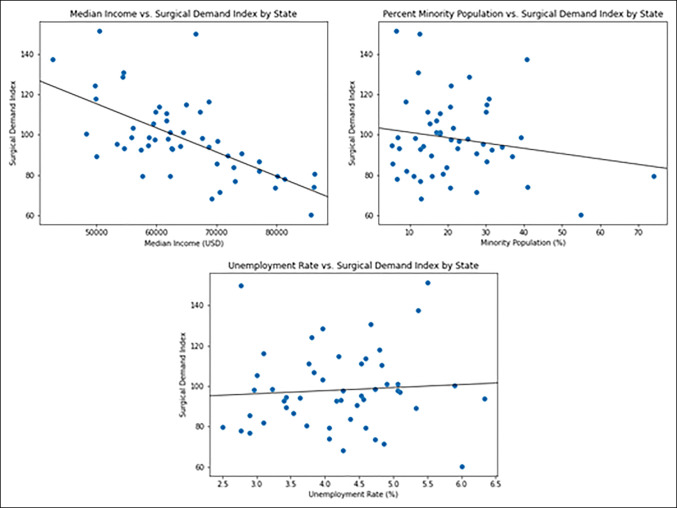
Diagram showing the scatter plot with least squares polynomial fit lines between each regressor: median income (top left), percent minority population (top right), and unemployment rate (bottom) and SDI. Ordinary least squares analysis (coefficient = −0.001, r2 = 0.42) revealed a significant effect of average income on SDI (*P* < 0.001), but no effect of percent minority population (*P* = 0.74) and unemployment rate (*P* = 0.37). SDI = surgical demand index

The analysis of variance results revealed a difference between geographic regions (*P* = 0.005). The pairwise Mann-Whitney results as given in Table 2 show that the South and Midwest were not significantly different from each other, but each had a higher average SDI when compared with the Northeast and the West. Similarly, the Northeast and West were not significantly different from each other, but each had a lower average SDI when compared with the South and Midwest. Linear regression analysis revealed a significant effect of median income on SDI (coefficient = −0.001, *r*^2^ = 0.42, *P* < 0.001), but no effect of percent minority population (*P* = 0.74) and unemployment rate (*P* = 0.37) (Figure 3).

**Table 2:** Mann-Whitney *U* Test Results of Mean SDI Between Each Geographical Region

*P*	Northeast	Midwest	South
Midwest	0.005*	—	—
South	0.025*	0.491	—
West	0.231	0.002*	0.015*

SDI = surgical demand index

* Significant.

## Discussion

This study assessed the surgical demand for elective orthopaedic procedures in each state by collecting GETH data and correlating that information with the number of orthopaedic surgeons in that area divided by the population of that area. An SDI was thus created that could be compared between states. It was hypothesized that the states with the lowest average annual income would have the greatest SDI, and the results of this study support that hypothesis. The relationships between SDI and percent minority population and unemployment rate were also explored; although there was a national 21% minority population and a 4% unemployment rate on average across 2016 to 2018, neither factor markedly affected SDI. The reason behind the lack of a relationship between unemployment status and minority population is left to conjecture and additional research. Notably, the SDI for each state is an arbitrary value and is designed to represent the demand-to-supply ratio of a state relative to other states.

One interesting application of these results is for re-evaluating the states which are most in need of orthopaedic surgery outpatient care. As reported in the AAOS 2018 census, Mississippi, Texas, and Nevada rank 50, 49, and 58 (ranking not including District of Columbia) in the number of orthopaedic surgeons per 100,000 population and therefore would be considered the top three most underserved states. Taking these data at face value imply that health policy legislators and orthopaedic care providers should focus on increasing access in these states. As a metric, however, orthopaedic surgeon density is incomplete because it only assesses surgeon supply without considering patient demand. Using the SDI, which accounts for both factors, Texas and Nevada drop from ranks 2 and 3 to ranks 29 and 28, respectively, in demand to supply disparity, with SDIs that fall below the mean SDI across the United States. This revelation suggests that the lower densities of orthopaedic surgeons in these states are in fact adequate for meeting the demands of the state populations regarding outpatient orthopaedic procedures. Instead, the focus should be placed on West Virginia, North Dakota, and Mississippi, which have the three highest SDIs. These results can be further leveraged by physicians looking to start private practices in underserved areas, hospital systems seeking to quantify patient demand in their states, and policymakers seeking data to make evidence-based decisions that affect both state and federal health systems.

One unique aspect of this study is that the interest scores in this study were created using composite search queries consisting of multiple different terms that people may search for when researching outpatient orthopaedic procedures. In addition, these composite queries were made across multiple topics in orthopaedics, resulting in a more accurate and holistic gauge of public interest value than using a single term alone, as has been done in previous studies.^18^ Additional studies can be conducted using even more elaborate query terms, hopefully improving on this study by capturing more public interest.

The results of this study can only be appreciated in the setting of its limitations, of which there are many. One such limitation is the accuracy of GETH data; specifically, that it leaves out certain populations who do not actively use Google, or who use it in a way not captured by the search terms in this study. Some such populations include those who do not search for their symptoms before evaluation and non-English speakers. However, the majority of patients were shown to search for their symptoms before evaluation in one study, the majority of the country had internet access during the search window, was English literate during this study window, and uses the Google search engine for their search queries.^1,16,17^ As such, the effect of excluding this population is believed to be small and likely do not markedly reduce the accuracy of the results of this study. This fact, however, provides motivation for a future study to assess orthopaedic surgical demand across those populations who do not search online, or who use languages besides English. Another limitation is that the data on two states, Maryland and Montana, were unable to be collected using the GETH extraction tool. The exact reason behind this was unable to be determined by these investigators, but it is being explored further because the code for the extraction tool itself undergoes additional revisions. The use of the GETH Python API for data collection in those states provides cooperative data for those areas.

Despite these limitations, this study provides a novel method to quantify the discrepancies between supply and demand for orthopaedic outpatient care and exemplifies one way to apply the field of infodemiology to healthcare research. This paper serves as a model for other biomedical informatics researchers to incorporate large-scale public interest data into their models by leveraging the innovative GETH tool.

## Conclusion

The goal of this project was to create a SDI for elective orthopaedic procedures for each of the 50 United States to identify areas with greater need and assess three potential predictive factors. An SDI was successfully created by collecting Google search probability data from GETH and normalizing search interest by the density of orthopaedic surgeons based on census data from AAOS. The Midwest and South had higher regional demand than the Northeast and West, with West Virginia being the most in need and the District of Columbia being the least in need. Annual median income had a notable negative effect on SDI, while percent minority population and unemployment rate did not. The novel metric proposed in this study may assist in highlighting healthcare disparities in the United States, in regions such as the South and Midwest. The knowledge of which specific states are underserved or oversaturated will help inform action to better distribute physicians to areas of increased need. Future research on this topic may include verifying the SDI against preexisting healthcare utilization metrics, assessing the effect of additional healthcare disparity data such as percent uninsured or percent on Medicaid on the SDI, incorporating regional demographic data into the SDI such as stratification by states using the Affordable Healthcare Act Medicaid Expansion, increasing the geographic granularity of the SDI to the county level, and using monthly or weekly data to identify temporal trends in the SDI.
